# A brief review of the science behind the design of healthy and sustainable plant-based foods

**DOI:** 10.1038/s41538-021-00099-y

**Published:** 2021-06-03

**Authors:** David Julian McClements, Lutz Grossmann

**Affiliations:** Department of Food Science, University of Massachusetts, Amherst, MA USA

**Keywords:** Colloids, Gels and hydrogels

## Abstract

People are being encouraged to consume more plant-based foods to reduce the negative impacts of the modern food supply on human and global health. The food industry is therefore creating a new generation of plant-based products to meet this demand, including meat, fish, egg, milk, cheese, and yogurt analogs. The main challenge in this area is to simulate the desirable appearance, texture, flavor, mouthfeel, nutrition, and functionality of these products using healthy, affordable, and sustainable plant-derived ingredients, such as lipids, proteins, and carbohydrates. The molecular and physicochemical properties of plant-derived ingredients are very different from those of animal-derived ones. It is therefore critical to understand the fundamental attributes of plant-derived ingredients and how they can be assembled into structures resembling those found in animal products. This short review provides an overview of the current status of the scientific understanding of plant-based foods and highlights areas where further research is required. In particular, it focuses on the chemical, physical, and functional properties of plant ingredients; the processing operations that can be used to convert these ingredients into food products; and the science behind the creation of some common plant-based foods, namely meat, egg, and milk analogs.

## Introduction

The modern food and agricultural industries have produced a plentiful supply of safe, affordable, convenient, and tasty foods, contributing to a significant reduction in world hunger and malnutrition over the past century. But current food production practices are also linked to the high prevalence of some chronic diseases, as well as to appreciable environmental damage^1–3^. A higher quantity and enhanced quality of food are required to feed a global population that is growing and becoming wealthier^3^. The production of large quantities of animal products, such as meat, fish, egg, milk, and their derivatives, has been proposed to be a major factor contributing to the negative impact of the modern food supply on global environmental sustainability^1^. Rearing livestock for food typically leads to more pollution, as well as greater greenhouse gas emissions, water use, land use, and loss of biodiversity than growing plants directly for consumption^4^. It should be noted, however, that there are areas unsuitable for the production of agricultural crops that are suitable for the raising of animals as foods. Moreover, some studies have shown that switching to a more plant-based diet may result in a slight increase in overall water use and only a modest decrease in overall cropland use^4^. However, many people have ethical concerns about confining and slaughtering animals, which is motivating them to switch to a more plant-based diet^5,6^. Moreover, many consumers believe a plant-based diet is healthier than an animal-based one, which is driving changes in their eating behaviors^6^, but it is important to note that a plant-based diet is not necessarily better than an omnivore diet from a nutritional perspective^7^. Animal foods, such as meat, milk, and egg, often contain micronutrients that are lacking from an entirely plant-based diet, such as vitamin D, calcium, and zinc. For this reason, plant-based foods often need to be fortified with these micronutrients.

As a result of these environmental, ethical, and health concerns, the plant-based food sector is expanding rapidly to meet consumer demand^8^. This sector includes a range of products created as alternatives to those normally produced from animals, including milk, meat, fish, eggs, and products where they are used as ingredients (Table 1). Each product category is expected to have its own unique physical, functional, nutritional, and sensory attributes. The food industry must therefore identify appropriate combinations of ingredients and manufacturing operations to economically create these attributes in plant-based foods on a large scale. As a result, they need knowledge of the molecular and physicochemical properties of plant-derived ingredients, how they can be assembled into structures that mimic those found in animal products, and how these structures influence the physicochemical and organoleptic properties of the end product. Ideally, these plant-based products should also be designed to be healthy, which involves controlling their nutrient profile, digestibility, and bioavailability. In the case of plant proteins, it is important to ensure that they are able to provide the full complement of essential amino acids and that they are digestible^9^. A well-balanced essential amino acid profile can often be achieved by consuming a mixture of plant proteins from different sources, such as grains and legumes.

**Table 1:** Market value of plant-based food products in the United States (2019), growth over a two-year period (2017–2019), and market share (2019).

Category	Value ($)	Growth (%)	Share (%)
Milk	$2,016,540	14%	40.5%
Meat	$939,459	38%	18.9%
Meals	$376,972	26%	7.6%
Ice cream and frozen novelty	$335,549	34%	6.7%
Creamer	$286,662	93%	5.8%
Yogurt	$282,502	95%	5.7%
Butter	$198,359	15%	4.0%
Cheese	$189,099	51%	3.8%
Tofu and tempeh	$127,856	15%	2.6%
Ready-to-drink beverages	$122,276	39%	2.5%
Condiments, dressings, and mayo	$63,696	1.4%	1.3%
Dairy spreads, dips, sour cream, and sauces	$29,513	135%	0.6%
Eggs	$9,851	228%	0.2%
	**$4,978,587**	**29%**	**100%**

Adapted from Cross (2020)^8^.

The bold values show the total amount in each category.

## Plant-based ingredients

Initially, it is important to identify an appropriate blend of plant-derived ingredients to produce a specific plant-based food, such as a meat, fish, egg, or milk analog. These ingredients may be isolated nutrients (such as proteins, carbohydrates, fats, vitamins, or minerals) or complex whole materials (such as beans, peas, rice, wheat, mushrooms, etc.). These ingredients have compositions, structures, and physicochemical properties that are very different from those found in animal products. One of the major challenges is therefore to assemble these ingredients into animal product analogs. Sometimes plant-derived ingredients can be used as-is (e.g., mushrooms), but in other cases, they may have to be dissembled into specific structural elements before being reassembled into animal product analogs (e.g., soy proteins). A brief outline of some of the main plant-derived ingredients used to form plant-based foods is given here.

### Plant-based proteins

**Fig. 1:**
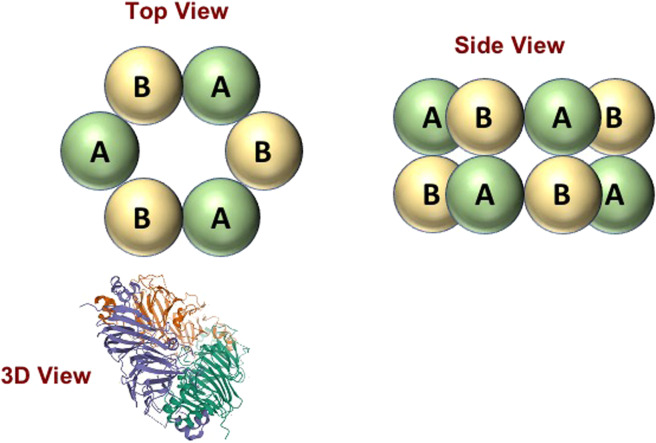
Globular plant proteins are often present as multimers linked together. The 3D view is for the soy glycinin hexamer, which is from the Protein Data Bank 1FXZ: Adachi, M., Takenaka, Y., Gidamis, A. B., Mikami, B., Utsumi, S. Crystal structure of soybean proglycinin A1aB1b homotrimer. *J. Mol. Biol.* **305**, 291–305 (2001). doi: 10.1006/jmbi.2000.4310.

**Fig. 2:**
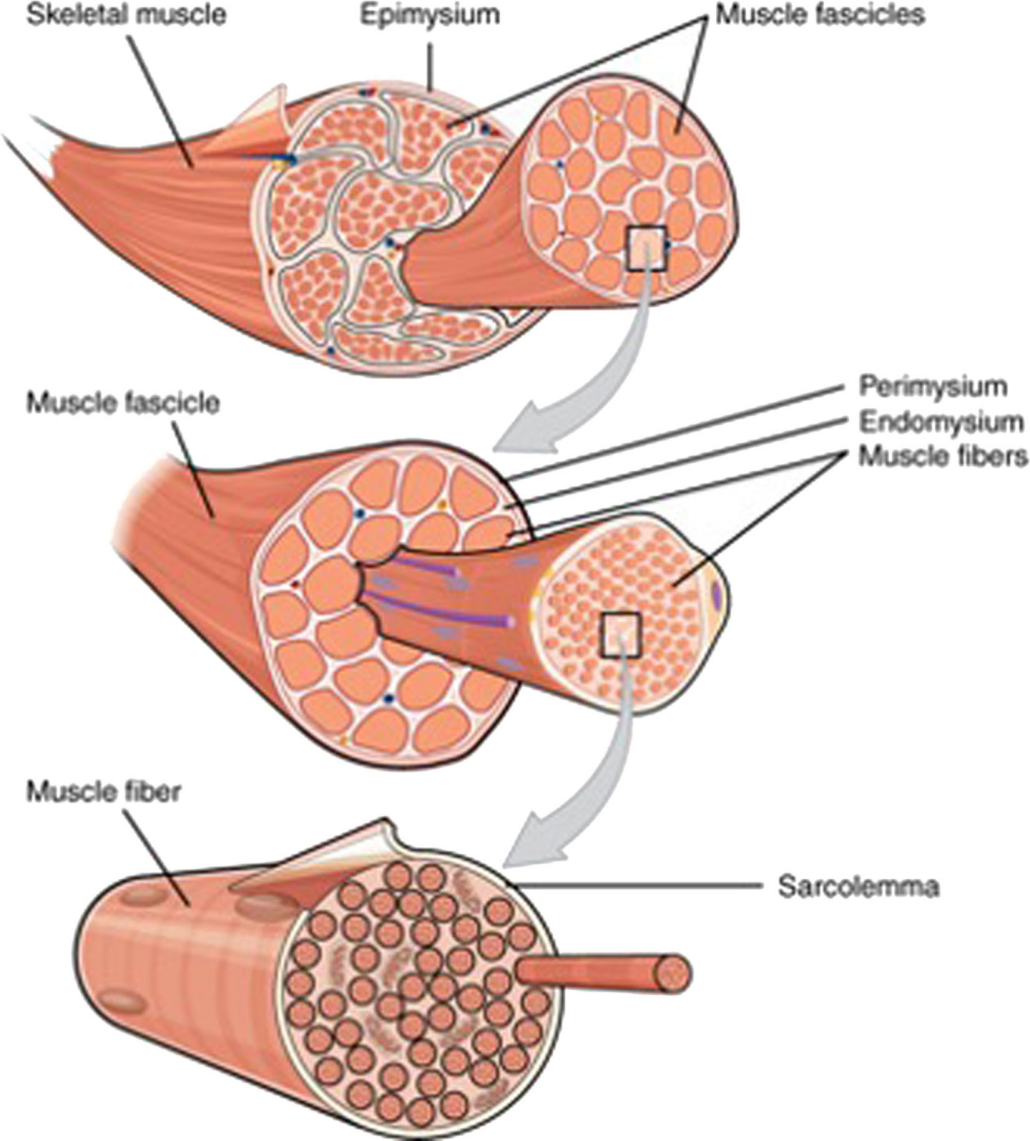
The muscles in meat have a complex hierarchical structure. The image of the meat structure used is from: OpenStax, CC BY 4.0.https://creativecommons.org/licenses/by/4.0, via Wikimedia Commons.

Plant proteins are commonly used in plant-based foods because of their versatile functional attributes, such as their ability to thicken, gel, emulsify, foam, and hold fluids^9,10^. In addition, they are an important source of essential amino acids. These proteins can be derived from various botanical sources, including soybeans, peas, faba beans, mung beans, lentils, algae, and microalgae, each with its own unique characteristics (Table 2). Most plant proteins have globular structures and are often present as complex multimers consisting of numerous different types of protein held together by physical and/or chemical bonds (Fig. 1). The functionality of these proteins depends on their biological origin, as well as any changes in their association and native states during isolation and purification. A major challenge in the plant-based food sector is the lack of plant proteins with consistent functional attributes. In the future, more research is required to identify appropriate botanical sources and isolation procedures for producing reliable functional ingredients. Another major challenge is to coax plant proteins into structural organizations that mimic those found in animal products (Fig. 2), thereby leading to similar physicochemical attributes.

**Table 2:** Molecular properties of selected plant and animal proteins.

	*M*_W_ (kDa)	pI	*T*_m_ (°C)
*Meat proteins*			
Collagen	300	5–8	62–67
Hemoglobin	67	6.8	67
Myoglobin	17	6.8–7.2	79
Actin	43	~5.2	70–80
Myosin	520	~5.3	40–50
Sarcoplasmic	20–100	Varies	50–70
*Egg proteins*			
Ovalbumin	45	4.6	85
Conalbumin	80	6.6	63
Ovomucoid	28	3.9	70
Ovoglobulins	30–45	5.5–5.8	93
Lysozyme	14.6	10.7	78
*Milk proteins*			
α_S1_−casein	23.6	4.6	–
α_S2_−casein	25.2	4.6	–
β−casein	24.0	4.6	–
κ−casein	19.6	4.6	–
β−lactoglobulin	18.4	5.4	72
α−lactalbumin	14.2	4.4	35 and 64*
BSA	66.3	4.9	64
*Plant proteins*			
Soy protein	150–380	4.5–5.0	80–93
Pea protein	50–360	4.5	75–79
Lentil protein	15–82	4.5	120
Chickpea protein	15–82	4.5	90
Lupin protein	150–216	4.5	79–101
Canola protein	14–59	4.5	84–102
Corn zein	14–27	6.4	89

*The lower and higher temperatures for alpha-lactalbumin are for the apo- (calcium free) and holo- (calcium bound) forms, respectively.

### Plant-based carbohydrates

Carbohydrates, such as sugars, oligosaccharides, or polysaccharides, can also be used as functional ingredients to assemble animal product analogs^11^. Plant-derived carbohydrates exhibit different molecular, physicochemical, functional, and biological properties depending on their biological origin and isolation procedures. These ingredients may be used to provide a variety of functional attributes in plant-based foods, including sweetness, thickness, gelling, emulsification, structure formation, stabilization, and fluid holding^12^. They may also be digestible or indigestible (i.e., fibers), as well as fermentable or non-fermentable, which impacts human nutrition and health. It is therefore important to select carbohydrate ingredients that provide the required quality and nutritional attributes in the end product. Polysaccharides are often used in combination with proteins to obtain desirable textural and sensory properties in plant-based foods *via* phase separation and interactive mechanisms^13^.

### Plant-based lipids

**Fig. 3:**
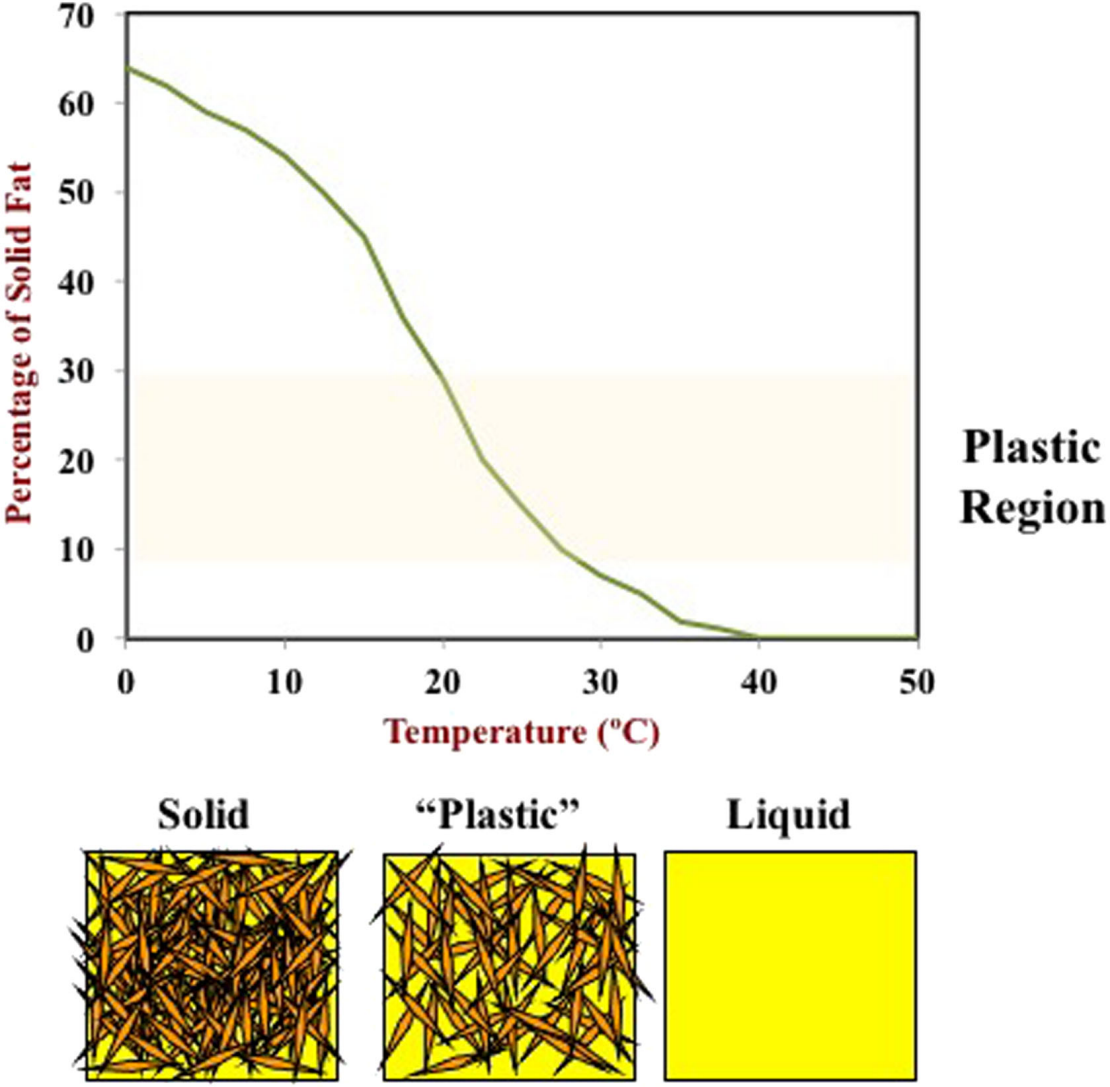
This figure shows the change in SFC with temperature (top), as well as the different crystal contents in lipids with temperature (bottom). The SFC-temperature profile of an edible fat determines its functionality.

Plant-based fats and oils can be economically extracted from various lipid-rich botanical sources, including algae, canola, coconut, cocoa, corn, flaxseed, olive, palm, safflower, soybean, and sunflower. For many applications, the ability of the triacylglycerols to form a 3D network of fat crystals is important, as it provides desirable textural attributes, such as the plasticity of butter and spreads (Fig. 3). Moreover, the change in the solid fat content with temperature plays a critical role in the functionality of many foods. This is particularly important when trying to mimic the behavior of animal fats with plant fats. The melting point of fats increases as the number of carbon atoms in the fatty acid chains increases or the number of double bonds decreases. The crystallization characteristics of fats are responsible for many of the desired quality attributes of animal products, such as butter spreadability, whipped cream foamability, cheese meltability, and ice cream hardness. For this reason, it is often important to simulate the crystallization characteristics of animal fats using plant-derived ones. This is often challenging because plant-derived fats contain more unsaturated fatty acids than animal fats and so tend to be more fluid-like at ambient temperatures. This problem can be overcome by increasing the degree of saturation of these fats using hydrogenation, but this may have adverse nutritional effects. As a result, food manufacturers often use naturally occurring high-melting plant-derived fats in their products, such as cocoa butter and coconut oil, but these also have high degrees of saturation that may have adverse health effects, such as an increased risk of heart disease^7^.

The type of fatty acids present in plant-derived fats and oils also influences their nutritional profile and oxidative stability. Fats and oils containing high levels of polyunsaturated fatty acids (PUFAs), particularly omega-3 ones (like flaxseed or algae oils), have been claimed to have beneficial health effects, such as the ability to reduce heart and brain diseases^14,15^. Although further studies are required to substantiate these claims using randomized clinical trials and meta-analysis. These fats may be used as an alternative to fish oils, which are rich in omega-3 PUFAs. Even so, it is important to prevent these PUFAs from oxidizing during storage and processing since this leads to the generation of undesirable off-flavors and toxic reaction products^16,17^. This may be achieved using numerous strategies including controlling temperature, oxygen, and light levels; reducing pro-oxidant contamination; incorporating antioxidants; utilizing chelating agents, or structuring approaches^18–20^. Utilization of these approaches will be important for creating the next generation of nutritionally-fortified plant-based foods.

### Other additives

The creation of high-quality plant-based foods also requires the utilization of various other additives, including colors, flavors, buffers, preservatives, and crosslinking agents^21^. Ideally, these ingredients should be natural botanical ingredients, like natural pigments (e.g., carotenoids, anthocyanins, and curcuminoids) or preservatives (e.g., essential oils or antimicrobial peptides).

## The science behind plant-based foods

In general, plant-derived ingredients are being used to create a wide range of food products to replace animal-based ones (such as meat, fish, eggs, and milk) or that normally require animal ingredients as key components (such as cheese, dressings, sauces, spreads, and yogurts) (Table 1). Here, we give a brief overview of the science and technology behind the formulation of the main categories of plant-based alternatives: meat, milk, and egg.

### Plant-based meat analogs

The recent commercial success of plant-based meat products, such as those produced by Beyond Meat and Impossible Foods, has had a profound impact on the modern food industry^21^. Indeed, the market for plant-based meats in the US was nearly $940 million in 2019, with a 38% increase from two years before (Table 1).

The food industry has been highly successful in producing high-quality analogs of comminuted meat products, such as burgers, sausages, nuggets, and ground meat since texturized vegetable proteins (TVPs) can be used to simulate their structures. However, it has proved much more challenging to create products that accurately mimic the properties of whole muscle tissue, which consists of muscle fibers, connective tissue, and adipose tissue organized into complex hierarchical structures (Fig. 2). The structural arrangement of these tissues plays a critical role in determining the physicochemical and sensory attributes of real meat products^22^.

**Fig. 4:**
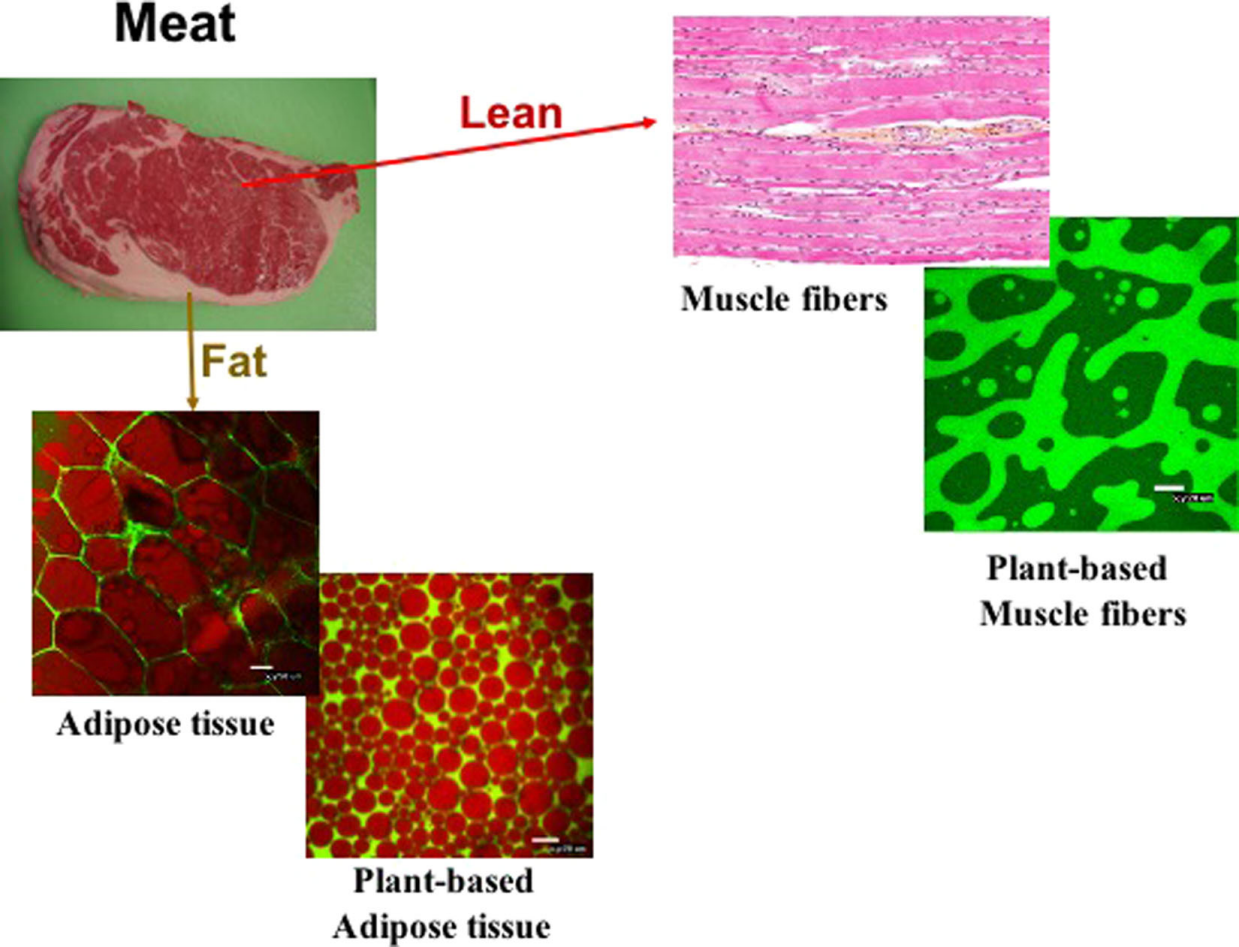
Soft matter physics is used to create meat-like structures from plant ingredients. The authors thank Xiaoyan Hu and Cheryl Chung (UMASS) for providing the images of adipose tissue and plant-based muscle fibers. The image of the muscle fibers is by Nephron and is licensed under CC BY-SA 3.0. The image of the raw beef steak is by Jellaluna and is licensed under CC BY 2.0.

The production of high-quality plant-based whole muscle analogs requires selecting the most appropriate ingredients and processing operations to simulate muscle fiber, connective, and adipose tissue (Fig. 4). Here, we highlight some of the key factors that should be considered when designing meat analogs that faithfully simulate the attributes of real meat. More details about this topic can be found in a number of recent review articles^23–25^. Ideally, meat analogs should reliably mimic the desirable characteristics of real meat products before, after, and during cooking. Meat analogs are mainly constructed from plant-derived macronutrients (fats, proteins, and polysaccharides), but also contain micronutrients and other additives, such as vitamins, minerals, colors, flavorings, binders, and preservatives^21^. The ingredients and processing operations used to produce these analogs must be optimized for each specific meat product being mimicked.

#### Appearance

The opaque nature of real meat can be simulated by including particles or fibers with dimensions (200–2000 nm) that scatter light strongly. The surface sheen of meat can be simulated by controlling the surface roughness and wetness of meat analogs. The analogs should have a wet smooth surface before heating leading to specular reflectance and a shiny look, but a rough dry surface after heating leading to diffuse reflectance and a matt look. The color of real meat is simulated by incorporating natural pigments that selectively absorb light at appropriate wavelengths. For instance, a beef analog should be pinky-red before cooking and brown after cooking. For some products, such as microwavable ones, it is only required to reproduce the brownish color of the cooked product.

Food companies have used various strategies to simulate the color of real meat in their plant-based alternatives. Beyond Meat^TM^ uses an extract from beet juice extract containing betalain (a natural pigment) to recreate the desirable color of meat. The betalain undergoes a chemical transformation when heated, causing it to turn from reddish-violet to orangey-yellow^26,27^. Impossible Foods^TM^ uses a plant-based heme protein, leghemoglobin, in their products. In principle, leghemoglobin can be extracted from the roots of soybeans, but in practice, it is more economically viable to generate it by microbial fermentation. Other natural pigments can be used either alone or in combination to create desirable meat-like color characteristics^28^.

#### Texture

It is possible to simulate the textural attributes of comminuted meat products (sausages, burgers, and nuggets) fairly accurately using TVPs, which has led to highly successful commercial plant-based products such as those from Impossible Foods^TM^ and Beyond Meat^TM ^^25^. It is much more challenging to simulate the delicate texture and mouthfeel of whole muscle products, like beef steaks, chicken breast, or pork chops because of their complex hierarchical structures (Fig. 2). A range of scientific and technological approaches are being explored for their potential in creating structures from plant-derived ingredients that simulate those found in real meat, with the ultimate aim of accurately mimicking their texture and mouthfeel^29^. These approaches can be grouped into two different categories that may be used separately or combined: physicochemical and processing approaches.

**Fig. 5:**
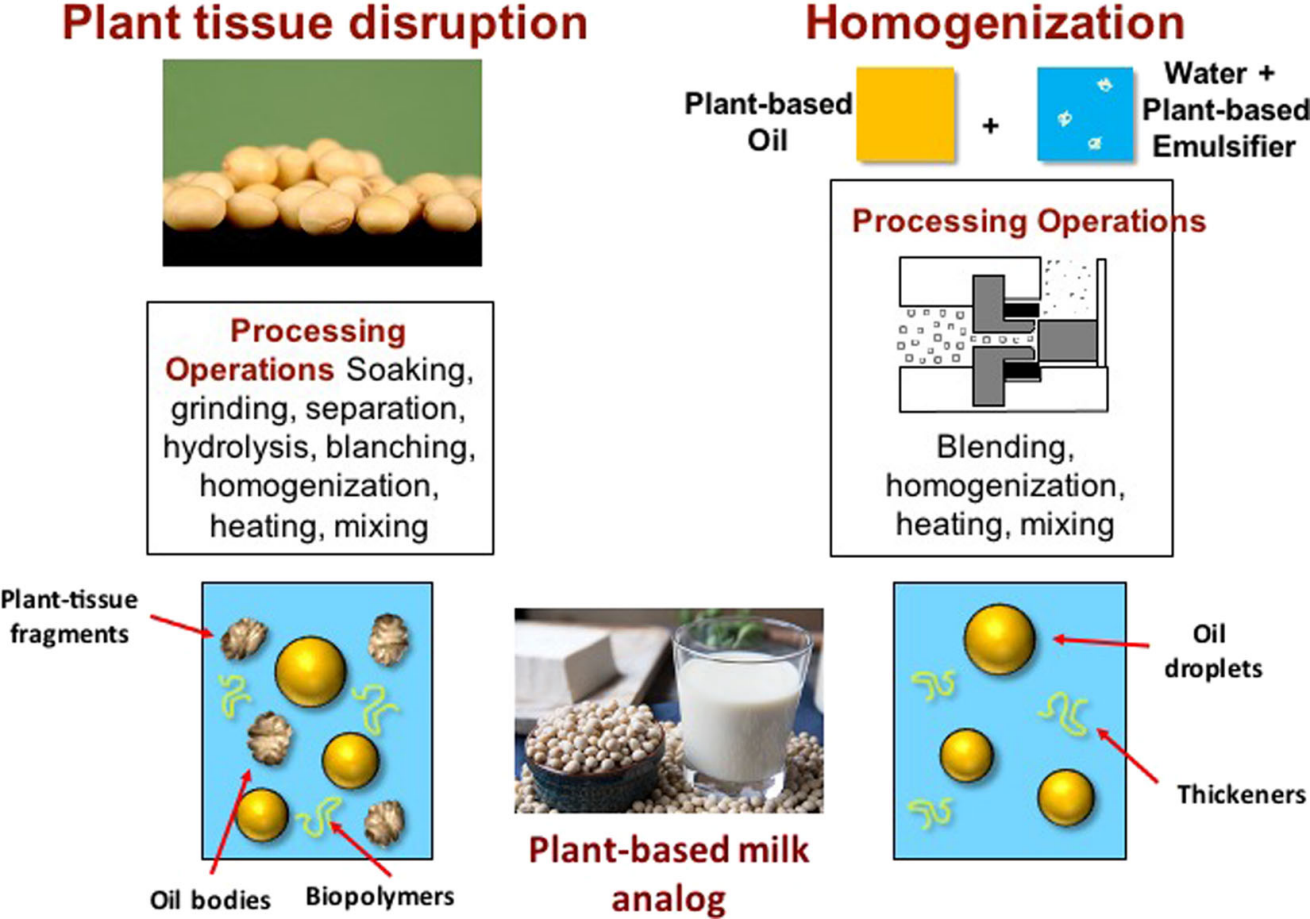
Plant-based milk can be produced by fragmentation or homogenization methods. Image of soybeans from CSIRO (CC BY 3.0). Image of “Soy Milk” by Kjokkenutstyr.net is licensed under CC BY-SA 2.0 (www.kjokkenutstyr.net).

Physicochemical approaches are based on controlling the molecular interactions and organization of plant-derived biopolymers to create meat-like structures^24,30^. Typically, a mixture of plant proteins and polysaccharides is used for this purpose. Appropriate mixtures of biopolymers can be made to phase separate by controlling the ingredient types and concentrations, as well as solution properties such as pH, mineral composition, and temperature (Fig. 4). The two main phase separation approaches involved are thermodynamic incompatibility and coacervation, which are based on inducing either repulsive or attractive interactions between the two types of biopolymers, respectively. This leads to the formation of a water-in-water (W/W) emulsion that contains two aqueous phases with different compositions. A mild shear force is then applied to the phase-separated biopolymer solution, resulting in the generation of fiber-like structures. These structures can then be locked into place by adding a suitable gelling agent or by changing the temperature (cooling or heating). This approach can be used to form fibrous structures that simulate some of the characteristics of those found in real meat, thereby leading to some similar physicochemical attributes (Fig. 5).

Plant-derived biopolymers can also be used to form meat-like structures using certain kinds of mechanical processing devices, such as extruders or high shear cells. As an example, protein-water mixtures are fed into an extrusion device, which mixes and shears them under high pressure and then extrudes them through a shaped die to form meat-like structures and textures^29,31,32^. Alternatively, these structures and textures can be formed by placing a mixture of proteins and polysaccharides into a specially designed cone-in-cone shear cell, which applies strong shear forces to the mixture by rotating one or both of the plates at a high speed. The biopolymer mixture can also be heated within the cell during the shearing process to promote protein unfolding and aggregation. As a result, the proteins organize into fiber-like structures that somewhat resemble the structure of meat fibers^13^. Extrusion methods are currently the most common processing method to create meat-like textures in commercial products, but the shear cell is also finding increasing use.

#### Cooking loss

An important attribute of real meat products is their ability to retain/lose fluids during cooking, as their fluid content impacts their look, feel, mouthfeel, and cooking properties. It is therefore important that meat analogs simulate the fluid-holding properties of real meat. Researchers have used fundamental physical chemistry models to identify the key factors impacting the fluid-holding properties of meat analogs: the interactions between the solvent and biopolymer molecules; the elastic modulus of the gel network formed by the biopolymer molecules; and, the osmotic pressure generated due to a concentration imbalance of mineral ions inside and outside the gel network^33^. The fluid holding properties of meat analogs can therefore be manipulated by altering biopolymer type, concentration, and crosslinking. In addition, the incorporation of polysaccharides can be used to improve the fluid holding properties^34^.

#### Flavor

Hundreds of aromatic molecules have been reported in meat products, but only some of these play a critical role in determining their characteristic flavor profiles^35^. The aroma profile depends on the type of meat and cooking method used. In cooked meat, the aromatic molecules are mainly the result of complex chemical reactions involving protein, carbohydrate, and lipid molecules, particularly Maillard and oxidation reactions. The taste of cooked meat depends on the balance of non-volatile molecules present that interact with umami, salt, sweet, bitter, and sour receptors in the mouth. These molecules may be present within the original raw animal flesh or they may be produced as a result of the cooking processes used.

Information about the most important flavor constituents within real meat products can be used to identify plant-based alternatives that provide meaty flavors in meat analogs. Impossible Foods uses soy leghemoglobin produced by fermentation processes to create “meaty” notes in their commercial meat analogs. The heme iron in leghemoglobin is exposed during cooking, thereby promoting oxidative reactions that generate aromatic compounds similar to those produced in real meat^36^. Mycoproteins, which are also produced using fermentation processes, are being utilized for their ability to produce meat-like aromas, tastes, and textures^37^. Algae and microalgae are being used in plant-based fish and other marine products because they provide seafood-like flavors^37^. Plant-derived materials can be used as precursors to form meaty flavors by carrying out controlled Maillard and oxidation reactions^38^. Research is also being carried out to reduce the undesirable flavors found in some plant-derived ingredients, e.g., the beany, earthy, astringent, or vegetative notes associated with chickpea, mung bean, or pea proteins^38^.

#### Nutritional profile

A major challenge when developing plant-based meat analogs is to match the nutritional profile of the original product. Meats contain high levels of protein, as well as essential micronutrients, such as zinc, iron, and vitamin B. Moreover, these micronutrients are often present in a highly bioavailable form within animal products. Consequently, it is important to design plant-based meat analogs that are enriched with bioavailable forms of these micronutrients. This can often be achieved using advanced encapsulation technologies, such as emulsions or nanoemulsions^39^.

### Plant-based milk analogs

Plant-based milk analogs are currently the most commonly consumed plant-based food products, contributing over 40% of the market sales in this sector (Table 1)^8^. The raw materials, processing methods, physicochemical properties, sensory attributes, and nutritional profiles of milk analogs products have been reviewed in a number of recent articles^8,40–43^. For this reason, only a short overview of these products is given here, with an emphasis on their fundamental properties.

#### Raw materials and production

Milk analogs are complex colloidal dispersions comprised of various kinds of particles, including oil bodies, fat droplets, protein aggregates, plant tissue fragments, and/or insoluble calcium carbonate particles, dispersed in an aqueous medium containing soluble proteins, polysaccharides, sugars, and salts^41^. Creating high-quality milk analogs, therefore, requires basic knowledge of colloid and interface science, such as particle reduction technologies, light scattering theory, and particle instability mechanisms. Milk analogs are typically created using two approaches: (i) plant tissue disruption; (ii) homogenization (Fig. 5)^41^. The first approach involves unit operations such as soaking, mechanical disruption, enzymatic hydrolysis, separation, formulation, homogenization, and thermal treatment to break down plant materials (such as soybeans, flaxseeds, almonds, or coconut flesh) into small particles. The second approach involves blending isolated plant-based ingredients (e.g., oils, emulsifiers, and thickeners) with water followed by homogenization and thermal treatment to produce an emulsion containing small droplets^41^. These processes must be carefully controlled to create stable milk analogs with the appropriate physicochemical, sensory, and functional attributes. Gravitational separation and aggregation can be inhibited by ensuring all the particles are sufficiently small (<500 nm), which can be achieved using appropriate chemical, enzymatic or mechanical size-reduction methods. Plant-based stabilizers, such as emulsifiers or thickening agents, may also be included to improve emulsion formation and stability. Plant-based emulsifiers include surface-active proteins (e.g., soy, pea, fava bean, and lentil proteins), polysaccharides (e.g., modified starches), phospholipids (e.g., soy and sunflower lecithin), or surfactants (e.g., quillaja and tea saponins)^44^. Plant-based thickening agents may be added to modify the textural characteristics or inhibit particle separation, which is usually polysaccharides like pectin, locust bean gum, gellan gum, starch, methylcellulose, carrageenan, and alginate^45^. The ingredients and processing operations used are optimized to create milk analogs that mimic the desirable properties and functional performance of cow’s milk^41^. Milk analogs may also be fortified with micronutrients to provide nutrients that may be deficient in plant-based diets, such as vitamin D, vitamin B_12_, and calcium^40^.

#### Appearance and sensory

A creamy appearance can be achieved in milk analogs by controlling the concentration and size of the colloidal particles they contain, such as oil bodies, fat droplets, and tissue fragments. Their lightness increases with increasing particle concentration and when the particles have similar dimensions to light waves (380–780 nm). The inherent color of milk analogs depends on the type and concentration of natural pigments they contain^46^. To achieve a desirable appearance it is often necessary to add or remove certain natural pigments.

The sensory attributes of cow’s milk are difficult to recreate because it has a bland but characteristic flavor profile, with over 100 volatile compounds typically present^47,48^. In contrast, milk analogs contain flavors arising from the plant’s raw materials, as well as generated during processing and storage. For instance, soymilks often have a beany flavor, whereas hazelnut milk has a nutty flavor^43^. Moreover, phytochemicals such as phenols and glucosinolates may introduce off-flavors, such as bitter, earthy, or vegetative notes^38^. Researchers are therefore developing new plant breeds and new processing methods to reduce off-flavors in milk analogs, including blanching and fermentation^49^.

#### Nutritional profile

The nutritional profile of plant-based milk products is often inferior to that of real milk^40^. Cow’s milk naturally contains high levels of vitamin A and calcium, which may be lacking in a plant-based diet. This problem can be overcome by using advanced encapsulation technologies to fortify plant-based milk with bioavailable forms of these micronutrients^40^.

### Plant-based egg analogs

Whole hen’s eggs are mainly comprised of water (75%), proteins (12%), and lipids (12%), and contain a diverse range of constituents that contribute to their various functional applications in foods, such as emulsification, foaming, water holding, and gelation^50^. As a result, they are versatile ingredients that can be used in many different foods, including alone (boiled, scrambled, poached, or fried eggs) or as a critical part of other foods (like mayonnaise, dressings, baked goods, and desserts). Ideally, plant-based egg analogs should simulate these desirable physiochemical and functional attributes. One of the most important functional attributes is the ability to undergo a sol–gel transition when heated under similar cooking conditions as used for real eggs. Ideally, the globular plant proteins used in egg analogs should therefore have a denaturation temperature in the same range as real egg proteins (i.e., around 63–93 °C), but many plant proteins only denature at higher temperatures (e.g., around 90 °C for soy glycinin^51^). As a result, higher temperatures or longer heating times are often required to achieve the same structure formation and textural attributes as real eggs. Instrumental methods like differential scanning calorimetry and dynamic shear rheometry can be used to provide information about protein denaturation and gelation temperatures. Typically, it is important that the plant proteins used are in a native state prior to heating, which means their isolation conditions must be carefully controlled. The nature of the gels formed depends on protein type (e.g., soybean, pea, chickpea, bean, and sunflower), protein concentration, and environmental conditions (e.g., ionic strength, pH, and thermal history), which should therefore all be carefully controlled^52^. In some applications, the plant-based ingredients in egg analogs should also exhibit good emulsifying properties, such as in mayonnaise or dressings. Plant proteins or phospholipids used for this purpose should typically be soluble in water, capable of adsorbing to oil droplet surfaces, and able to stabilize oil droplets from aggregation. In some cases, other plant-based ingredients may also be required to prevent destabilization of the product, such as thickening agents that inhibit gravitational separation. The yellowish appearance of egg yolks may be achieved by adding natural pigments (such as curcumin or carotenoids), while an appropriate flavor profile may be achieved by adding natural flavors, herbs, or spices.

Many different egg analogs have been developed over the years, with JUST Egg^TM^ (www.ju.st) being one of the most successful recently. Two products are currently on the market from this company: (i) *fluid eggs* intended to prepare scrambled eggs or omelets; (ii) *frozen egg slices* that can be heated and used in breakfast sandwiches. Mung bean protein and emulsified canola oil are two of the main components of these products. The proteins unfold and aggregate during cooking leading to a gel-like texture. The canola oil droplets contribute to the opaque appearance, textural attributes, flavor profile, and mouthfeel of the final product. These products also contain transglutaminase, an enzyme that crosslinks the proteins, thereby increasing the gel strength and water holding capacity so as to better mimic real egg^53^. The yellowish color of eggs is mimicked in these products by adding turmeric, which contains curcumin. Other functional ingredients are also added to more closely simulate the properties of real eggs, including thickeners/stabilizers (e.g., corn starch and gellan gum), seasonings (e.g., garlic powder, onion powder, sugar, and salt), buffering salts (e.g., bicarbonates, citrates, or phosphates), and preservatives (e.g., nisin). In the future, more research is still required to improve the functional versatility of egg analogs and to enhance their nutritional profiles.

#### Nutritional profile

The nutritional profile of plant-based eggs is often worse than that of real hen’s eggs. Hen’s eggs naturally contain a variety of vitamins and minerals that are not commonly found in a plant-based diet. For this reason, it is often important to fortify plant-based egg products with bioavailable forms of these micronutrients, which often require the utilization of advanced encapsulation technologies.

## Conclusions and future directions

Recent reports suggest that human and global health would be greatly improved by replacing animal-based foods (such as meat, fish, eggs, milk, and their products) with plant-based alternatives. This transition would be facilitated by the availability of more plant-based foods that are affordable, convenient, sustainable, nutritious, and tasty. Consumers would then find it easier to change their dietary habits and adopt a more healthy and sustainable diet. There are, however, various hurdles that need to be addressed to achieve this goal:

- *Consumer-based hurdles*: Improved knowledge of the behavior of consumers is needed to create effective approaches to encourage them to try, like, and adopt plant-based foods. There has already been a considerable amount of consumer research carried out for certain kinds of plant-based products^54–56^. However, more research is required to develop effective materials to educate consumers about the potential benefits and drawbacks of consuming plant-based foods so they can make informed choices.
- *Technological-based hurdles*: The creation of plant-based foods is being held back by a lack of high-quality plant-derived ingredients, particularly proteins, as well as large-scale manufacturing processes to convert these ingredients into desirable end products. In particular, it is still challenging to create analogs of whole muscle meat, fish, yogurt, and cheese because of their complex structural hierarchies. Consequently, more research is required to understand the relationship between the structure and properties of plant-based ingredients and their ability to form high-quality meat, fish, egg, or dairy analogs people want to consume.
- *Commercial-based hurdles*: The commercialization of plant-based foods is being held back by a lack of knowledge about the relative advantages and disadvantages of different plant-derived ingredients and manufacturing processes, as well as of safety concerns (such as allergenicity), regulations in different countries, and supply chain issues. Increased knowledge about these issues would help companies to successfully enter the plant-based food market.
- *Social-based and economic-based hurdles*: Changes in government policies, such as taxation, incentives, and educational programs, would facilitate the transition to a more plant-based diet. However, improved knowledge about the social, economic, environmental, and health implications of replacing animal products with plant-based ones is still required to craft and implement these policies.

In the future, it will be important for governments, industries, and non-profit organizations to support efforts to obtain this information, thereby facilitating a more rapid transition to a healthy and sustainable plant-based diet. It should also be noted that many plant-based foods are highly processed and contain numerous additives, which is undesirable to many consumers. Consequently, there is a need for more research on the development of processed plant-based foods that contain fewer ingredients and involve less processing. In addition, it is often assumed that plant-based foods are healthier than animal-based ones. But this is often not the case. More research is required to ensure that plant-based foods are carefully designed to ensure that they have beneficial nutrient profiles and that the nutrients are in a bioavailable form.

## Data Availability

Data sharing not applicable. This is a review article and no new datasets were generated or analyzed during this study.
